# Discovery and validation of programmed cell death–associated key biomarker genes in ischemic stroke via ssGSEA/WGCNA and LASSO–SVM-RFE

**DOI:** 10.3389/fmolb.2026.1844734

**Published:** 2026-07-16

**Authors:** Qi Jia, Pengtao Zhang, Qianqian Liu, Enze Sang, Zhengwen Chen, Junjie Shao, Yuhao Ding, Faguang Bai, Qingfeng Huang

**Affiliations:** 1 Neurointerventional Center, Affiliated Hospital of Nantong University, Nantong, China; 2 Neurological Disease Center, Affiliated Hospital of Nantong University, Nantong, China; 3 Medical School of Nantong University, Nantong, China; 4 Department of Neurosurgery, Affiliated Hospital of Nantong University, Nantong, China; 5 Department of Neurosurgery, The Affiliated Kizilsu Kirghiz Autonomous Prefecture People’s Hospital of Nanjing Medical University, Artux, Xinjiang, China

**Keywords:** biomarkers, ischemic stroke, machine learning, programmed cell death, ssGSEA, WGCNA

## Abstract

**Background:**

Ischemic stroke (IS) currently lacks well-characterized peripheral-blood biomarkers that capture early, pathway-level biology. Programmed cell death (PCD) pathways may shape post-stroke neuroinflammation and could yield clinically informative transcriptional signatures.

**Methods:**

Public cohorts (GSE16561 discovery; GSE58294 external test) were analyzed. We quantified sample-level pan-PCD activity using ssGSEA based on a curated PCD gene set,identified PCD-associated modules via WGCNA, and intersected with limma-derived DEGs. Two complementary machine learning (LASSO and SVM-RFE) were used to select compact candidate biomarkers. Diagnostic performance was evaluated by ROC analysis. Immune infiltration was inferred by ssGSEA (28 immune signatures) and correlated with candidate genes. Drug candidates were prioritized using Enrichr/DSigDB and explored by molecular docking. *In vivo* validation in a rat MCAO model was additionally performed at the brain-tissue level.

**Results:**

A pan-PCD score was higher in IS than controls and guided WGCNA to a PCD-associated module. Intersection with DEGs yielded 58 PCD-related genes. LASSO and SVM-RFE converged on three biomarkers—CREBBP, ANTXR2, and ARG1. These genes showed consistent discriminative performance in both discovery (AUCs: 0.937–0.981) and external test cohorts (AUCs: 0.656–0.931) and were associated with neutrophil-skewed immune infiltration. *In vivo* validation in a rat MCAO model confirmed upregulation of all three genes in ischemic brain tissue. Enrichr/DSigDB prioritization and docking highlighted papaverine (CREBBP) and trichostatin A (ANTXR2) as plausible leads.

**Conclusion:**

An integrative network–ML framework delineated a peripheral-blood pan-PCD–related transcriptional pattern in IS and prioritized three biomarkers with consistent diagnostic performance and a neutrophil-skewed immune context. The exploratory pathway–gene–drug framework proposed here nominates testable compounds and provides a basis for prospective multi-cohort validation and mechanistic studies.

## Introduction

Ischemic stroke (IS) remains a leading cause of death and long-term disability worldwide (GBD 2021 Stroke Risk Factor Collaborators, 2024), with the absolute number of cases and years of life lost continuing to climb, particularly in aging and cardiometabolic populations (Feigin et al., 2025; Martin et al., 2024). Although endovascular thrombectomy and intravenous thrombolysis have transformed acute care, many patients present beyond treatment windows or achieve incomplete clinical benefit despite angiographic success (Wang et al., 2025; Hong et al., 2025). Beyond the initiating occlusion, a rapidly evolving neuroimmune cascade spans the hyperacute to subacute phases: neutrophils, monocytes/macrophages, microglia, and T-cell subsets drive cytokine/chemokine flux, oxidative bursts and neutrophil extracellular traps (NETs), complement activation, endothelial dysfunction, and blood–brain barrier breakdown, while also contributing to debris clearance and remodeling (Chou et al., 2023; Fang et al., 2024; Alsbrook et al., 2023). Converging evidence implicates multiple programmed cell death (PCD) modalities—including apoptosis, pyroptosis, ferroptosis, and necroptosis—as upstream regulators of neuronal/glial fate and modulators of immune–glia crosstalk that exacerbate excitotoxicity, lipid peroxidation, mitochondrial dysfunction, and barrier permeability (Qian et al., 2020; Guo et al., 2025; Yang, 2022; Xiong et al., 2026). These coordinated processes leave reproducible transcriptional footprints detectable in peripheral blood, opening a window for minimally invasive, pathway-level biomarkers to refine diagnosis, enable risk stratification, and inform mechanism-anchored therapies.

Despite mounting mechanistic insight into PCD–immune crosstalk in IS, most human transcriptomic studies interrogate single pathways or isolated genes, limiting generalizability across platforms, tissues, and clinical contexts (Gong et al., 2023; Rust, 2023). It remains unclear whether a unified, quantifiable “pan-PCD” transcriptional program in blood can stratify patients, mirror immune-microenvironment patterns, and nominate actionable targets (Stamova et al., 2026; Qi et al., 2023). Methodologically, many efforts rely on one analytic axis without combining sample-level pathway scoring, network approaches that preserve co-regulation, and orthogonal machine-learning feature selection to yield sparse yet stable signatures with external validity (Liu et al., 2025; Zhao et al., 2024). Translation often stalls at candidate lists; connections to drug repurposing are inconsistently grounded in modern gene–drug resources or structural plausibility, despite increasing availability of enrichment knowledge-graphs and drug-signature resources.

The rapid development of bioinformatics methods enables researchers to efficiently analyze the expression data of various disease-related genes, gain a deeper understanding of the pathological biological mechanisms of diseases from a genetic perspective, and play a key role in revealing the relationship between genes and diseases (Gao et al., 2026; Zhao et al., 2026; Chi et al., 2025; Huang et al., 2025). The lower the binding energy, the higher the binding stability of the interaction between a molecule docking target and a predicted drug (Tripathi et al., 2013; Du et al., 2020). Therefore, here, across multiple GEO cohorts, we implemented an integrative framework that quantified pan-PCD activity (ssGSEA), mapped aligned co-expression modules (WGCNA), distilled compact biomarkers with complementary ML pipelines, linked signatures to immune infiltration, and bridged discovery to therapeutics—prioritizing CREBBP, ANTXR2, and ARG1 as diagnostic candidates and nominating small-molecule leads for hypothesis generation.

## Materials and methods

### Data collection and preprocessing

Gene-expression profiles and annotations for GSE16561 and GSE58294 were retrieved from GEO (https://www.ncbi.nlm.nih.gov/geo/). GSE16561 (39 ischemic stroke, 24 controls) served as the discovery cohort; GSE58294 (69 cases, 23 controls) served as the external test cohort. Series matrix files and annotations were used with uniform within-cohort preprocessing (log2 transformation where required; collapsing probes to gene symbols by averaging duplicates); all datasets were de-identified and publicly available.

### Source of PCD–related genes

The PCD gene set used in this study was derived from a previously published resource (Zou et al., 2022). We adopted the curated, high-confidence PCD-related genes reported therein as our initial list. Gene symbols were harmonized to current HGNC standards, duplicates were removed, and only genes present in the analyzed datasets were retained for downstream scoring and modeling.

Although the included PCD modalities (e.g., apoptosis, pyroptosis, ferroptosis, necroptosis) have distinct molecular executioners and tissue-/time-specific roles, accumulating evidence indicates substantial cross-talk and mutual amplification among these programs under ischemic stress, and aggregated “pan-PCD” or “multi-death” scoring schemes have been used to summarize the combined transcriptional burden of cell-death machinery in human disease cohorts (Qian et al., 2020; Guo et al., 2025; Zou et al., 2022). In the present study, the pan-PCD score is therefore intended as a high-level summary index of overall PCD-related transcriptional activity in peripheral blood, rather than as a measure of any single death pathway; pathway-specific dissection is reserved for future work with higher-resolution data.

### Single-sample gene set enrichment analysis (ssGSEA)

We quantified PCD activity per sample using ssGSEA. For each dataset independently, genes were rank-ordered within each sample, and an enrichment score was computed as the difference between the empirical cumulative distributions of the PCD gene set and the remaining genes. The resulting ssGSEA score represents the coordinated up- or downregulation of the PCD program in that sample. Scores were computed separately within each cohort and subsequently standardized (z-score) for downstream correlation and modeling.

### Differential expression analysis

Differential expression was evaluated using empirical Bayes–moderated linear models implemented in the limma R package on gene-level expression matrices (Liu et al., 2021). For the case–control contrast, we computed log2 fold changes (log2FC), P values, and adjusted P values with Benjamini–Hochberg correction for multiple testing. Differentially expressed genes were defined by adjusted P < 0.05 and |log2FC| > log2 (1.5). Genes meeting these criteria were carried forward to downstream enrichment, network, and modeling analyses.

### WGCNA

We performed WGCNA on the GSE16561 dataset to identify co-expressed gene modules and examine their associations with study traits/pathway phenotypes (Han et al., 2021). Samples were first hierarchically clustered to screen for outliers. A scale-free topology criterion (target fit R2 > 0.85) guided soft-threshold selection; pickSoftThreshold () was used to determine β = 5. Based on this β, expression matrices were transformed into adjacency matrices and then into a topological overlap matrix (TOM). Genes were clustered in the TOM space using average-linkage hierarchical clustering, and initial modules were defined via dynamic tree cutting with deepSplit = 2 and minModuleSize = 80. Module eigengenes (MEs) were computed and similar modules were merged using mergeCutHeight = 0.25. Module–trait relationships (including PCD ssGSEA score and clinical grouping) were subsequently evaluated using ME-based correlations for downstream analyses.

### Immune infiltration

Immune cell infiltration was quantified using ssGSEA with 28 immune-cell gene signatures curated by Charoentong et al. (Charoentong et al., 2017). Within each cohort, the GSVA R package (method = “ssgsea”) was applied to compute enrichment scores per sample for each of the 28 signatures (Hänzelmann et al., 2013). Scores were standardized within cohort (z-scores) and used for downstream correlations with PCD scores, candidate gene expression, and module eigengenes.

### Molecular docking

This study evaluates the binding energy and interaction mode between candidate drugs and targets at the atomic level using molecular docking technology, in order to explore the potential of targets as therapeutic drugs. The two-dimensional structure of small molecule ligands was obtained from the PubChem database, while the three-dimensional structure of target proteins was retrieved from the PDB protein database. Subsequently, Autodock Vina software was used to perform molecular docking analysis on the selected drugs and their corresponding target proteins.

### Rat MCAO model (koizumi method)

Left middle-cerebral-artery occlusion (MCAO) was induced in adult rats using the Koizumi intraluminal filament method with permanent suture placement (no reperfusion). Briefly, a 4–0 rounded-tip nylon filament was advanced approximately 18 mm up the internal carotid artery to occlude the origin of the middle cerebral artery; sham-operated animals underwent the same surgical procedure without filament advancement. Twenty-four hours after occlusion, the ipsilateral fronto-parietal cortex was harvested for RNA extraction and downstream RT-qPCR analysis. This animal experiment was conducted as an exploratory, hypothesis-supporting cross-species component of the study rather than as a quantitative validation cohort, and was therefore performed at a small scale. All animal procedures were approved by the Animal Ethics Committee of Nantong University Affiliated Hospital (Approval No. P20250218-023) and complied with institutional guidelines for the care and use of laboratory animals.

### RT-qPCR

One microgram of total RNA was reverse-transcribed with PrimeScript RT kit (Takara). The resulting cDNA was amplified in triplicate with TB Green Premix II and gene-specific primers on a CFX96 system: 95 °C for 5 s, 60 °C for 30 s, 40 cycles. Relative expression was calculated by the Δ ΔCt method using GAPDH as reference and melt-curve verification.

### Statistical analysis

Two-sided tests were used with α = 0.05. Normality was checked (Shapiro–Wilk); group comparisons used Student’s t-test or Wilcoxon rank-sum, and categorical data used χ2 or Fisher’s exact tests. Associations among continuous variables were assessed by Spearman’s ρ with Benjamini–Hochberg FDR correction for multiple testing. Analyses were performed in R (≥4.2).

## Results

### Identification of PCD-related genes via WGCNA

**FIGURE 1:**
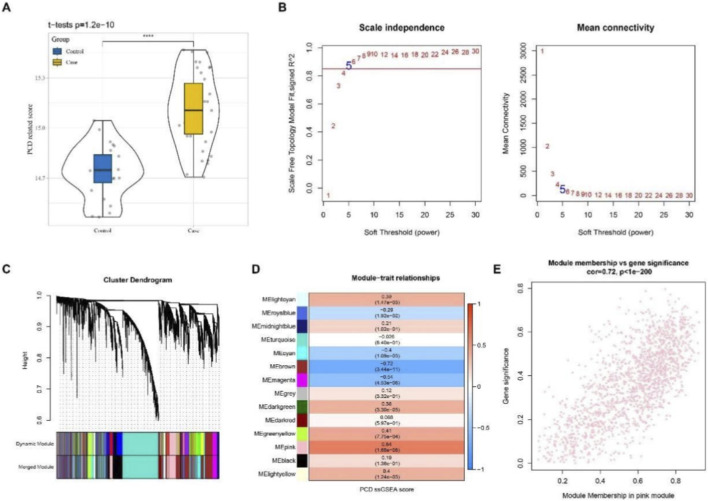
PCD scoring and WGCNA in GSE16561. **(A)** ssGSEA scores from the pan-PCD gene set. **(B)** WGCNA soft-threshold selection, β = 5. **(C)** Gene dendrogram (1 − TOM dissimilarity) with module assignment. **(D)** Module–trait (PCD ssGSEA) correlation heatmap. **(E)** GS–MM scatter plot for the MEpink module.

In the GSE16561 discovery cohort, ssGSEA-derived PCD scores were globally elevated in ischemic stroke (IS) samples relative to controls, indicating heightened PCD activity in disease (Figure 1A). Using these scores as the trait for WGCNA, the soft-thresholding power was selected as β = 5 by pickSoftThreshold (Figure 1B), yielding 14 co-expression modules (Figure 1C). Module–trait correlation analysis identified several modules significantly associated with the PCD score, with MEpink showing the strongest positive correlation (Figures 1D,E). Genes meeting GS > 0.3 and MM > 0.3 were designated as highly PCD-related candidates, resulting in 703 genes for downstream analyses.

### Identification of PCD-related differentially expressed genes

**FIGURE 2:**
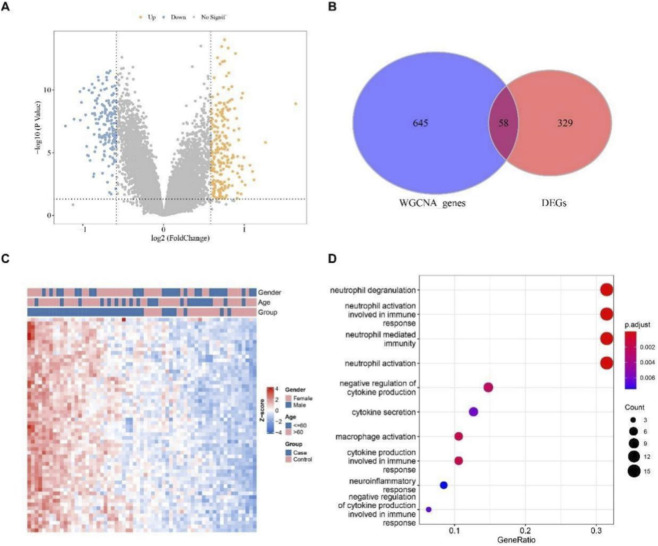
PCD-related DEGs and functional enrichment. **(A)** Volcano plot of DEGs (case–control). **(B)** Venn diagram of the intersection between DEGs and PCD-related module genes. **(C)** Heatmap of PCD-related DEGs. **(D)** GO–Biological Process enrichment bubble plot for PCD-related DEGs.

In GSE16561, differential expression between cases and controls was assessed using limma with thresholds of P < 0.05 and |log_2_FC| > log2 (1.5) (Figure 2A). Intersecting these differentially expressed genes with the WGCNA-derived PCD candidate set yielded 58 PCD-related differentially expressed genes (DEGs) (Figure 2B), whose expression patterns across groups are shown in the heatmap (Figure 2C). Functional enrichment with clusterProfiler (Figure 2D) indicated significant over-representation of neutrophil-associated immune processes, including neutrophil degranulation, neutrophil activation, and neutrophil activation involved in immune response.

### Machine-learning–based biomarker selection

**FIGURE 3:**
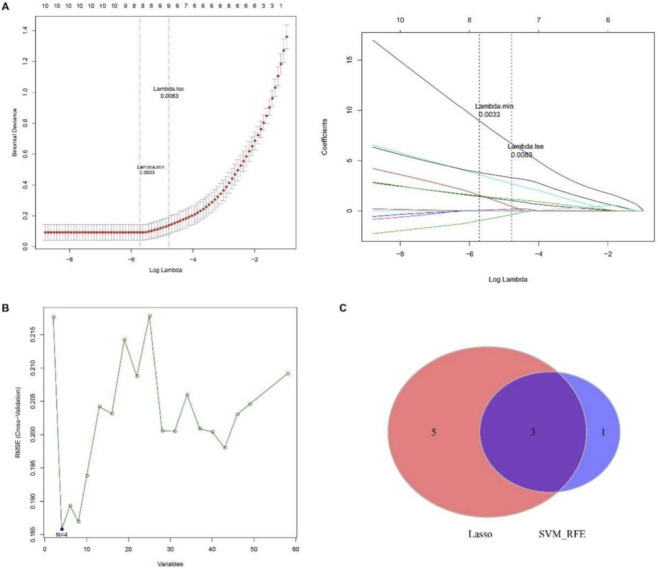
Machine-learning–based feature selection. **(A)** Left, LASSO cross-validation curve (log λ vs. deviance/df); right, LASSO coefficient paths (log λ vs. coefficients). **(B)** SVM-RFE curve showing feature count vs. cross-validated RMSE. **(C)** Venn diagram of overlapping features selected by LASSO and SVM-RFE.

From the PCD-related DEGs, we first applied LASSO regression using glmnet in R and retained the model at lambda. min = 0.0033 (Figure 3A) (Tay et al., 2023). We then performed recursive feature elimination (RFE) with the caret package, observing the lowest error when four features were included (Figure 3B). Intersecting candidates from both approaches yielded three robust biomarkers—CREBBP, ANTXR2, and ARG1 (Figure 3C).

### Diagnostic performance

**FIGURE 4:**
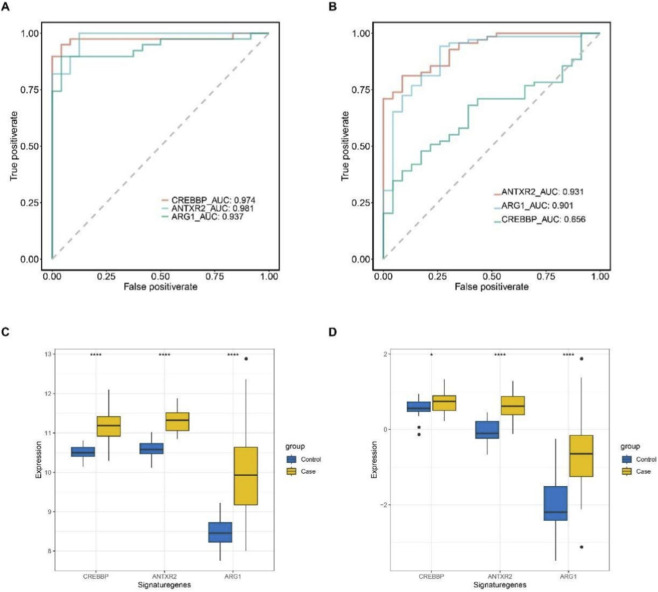
Diagnostic performance and expression of hub genes. **(A)** ROC curves for individual signature genes in the discovery cohort (GSE16561). **(B)** ROC curves for individual signature genes in the external test cohort (GSE58294). **(C)** Expression of hub genes (CREBBP, ANTXR2, ARG1) in cases vs. controls in the discovery cohort. **(D)** Expression of hub genes in cases vs. controls in the test cohort. *Statistical significance is indicated as follows: *P < 0.05, ****P < 0.0001.

In both the GSE16561 discovery cohort and the GSE58294 external test cohort, receiver operating characteristic (ROC) analyses were conducted for the selected biomarkers (Figures 4A,B). In the discovery cohort, CREBBP, ANTXR2, and ARG1 achieved AUCs of 0.974, 0.981, and 0.937, respectively. In the external test cohort, the corresponding AUCs were 0.656, 0.931, and 0.901, demonstrating consistent discriminative performance across independent datasets. Consistently, CREBBP, ANTXR2, and ARG1 exhibited higher expression in ischemic stroke samples than in controls (Figures 4C,D).

### Immune cell infiltration and correlations with biomarkers

**FIGURE 5:**
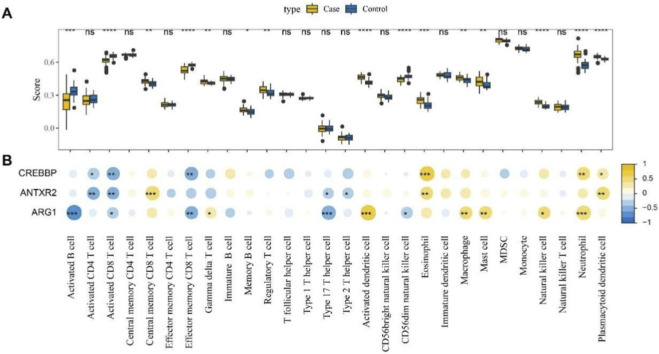
Immune infiltration and biomarker correlations. **(A)** Case–control differences in immune-cell infiltration. **(B)** Spearman correlation bubble plot between biomarkers (CREBBP, ANTXR2, ARG1) and immune-cell infiltration. *Statistical significance is indicated as follows: *P < 0.05, **P < 0.01, ***P < 0.001, ****P < 0.0001.

Using ssGSEA-derived scores for 28 immune-cell signatures, case–control comparisons revealed significant differences in infiltration across multiple cell types (Figure 5A). Spearman correlation analysis between the three biomarkers (CREBBP, ANTXR2, ARG1) and immune infiltration indicated positive associations with central memory CD8 T cells and neutrophils, and negative associations with effector memory CD8 T cells and type 17 T helper (Th17) cells (Figure 5B).

### Drug prioritization and molecular docking

**FIGURE 6:**
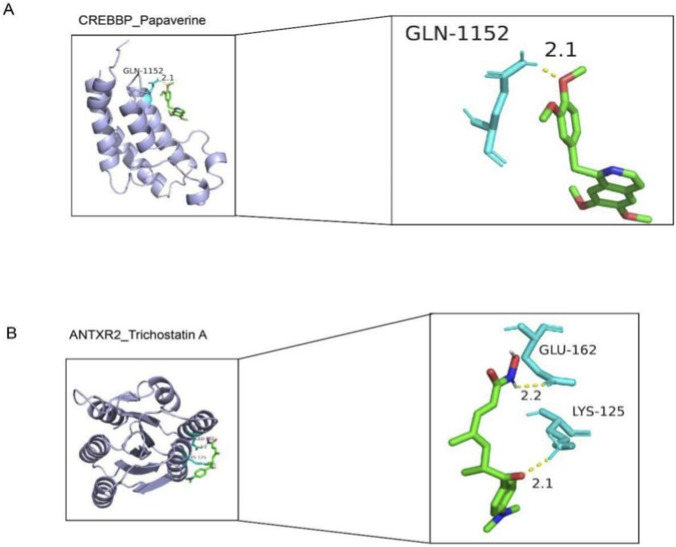
Molecular docking poses. **(A)** Docked pose of papaverine with CREBBP. **(B)** Docked pose ofTSA with ANTXR2. Insets: receptor protein in light blue, ligand in green, amino-acid residues in cyan; hydrogen bonds shown as yellow dashed lines with lengths in Å.

Drug-repositioning analysis using Enrichr/DSigDB with CREBBP, ANTXR2, and ARG1 as input yielded candidate compounds; papaverine and trichostatin A (TSA) were shortlisted based on rank and mechanistic plausibility. Ligand structures were retrieved from PubChem and energy-minimized; protein structures for CREBBP and ANTXR2 were obtained from the PDB and preprocessed. Semi-flexible docking was performed with AutoDock Vina, and poses were visualized in PyMOL. The CREBBP–papaverine binding pose is shown in Figure 6A, and the ANTXR2–TSA pose is shown in Figure 6B.

### Cross-species support from a rat MCAO model

**FIGURE 7:**
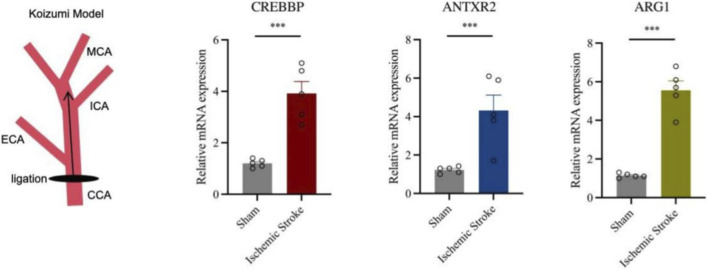
Validation of the Koizumi Model in rats. A schematic representation of the Koizumi Model is shown on the left, and RT-PCR results of the modeled brain tissue are shown on the right.

A rat ischemic stroke model was established using the Koizumi intraluminal-filament method, with the experimental group undergoing middle cerebral artery occlusion and the control group receiving sham surgery. Twenty-four hours after occlusion, RNA was extracted from ischemic ipsilateral cortical tissue and the expression of the three candidate genes was assessed by RT-qPCR. *In vivo* validation confirmed that CREBBP, ANTXR2 and ARG1 were all significantly upregulated in the ischemic brain tissue of MCAO rats relative to sham controls (Figure 7).

## Discussion

The pathological core of acute ischemic stroke (AIS) lies in ischemia–reperfusion–induced excitotoxicity, oxidative stress, and amplified inflammation, which together drive multiple forms of PCD and disrupt the neurovascular unit. These molecular events are not confined to the brain; they also leave a detectable “transcriptional footprint” in the peripheral immune system (Majumder, 2024; Qin et al., 2022). In recent years, several studies have shown that early peripheral blood transcriptomes can predict outcomes and delineate the temporal dynamics of the immune response, thereby supporting molecular subtyping and translational applications (Amini et al., 2023; Blase et al., 2025). Against this background, the present study integrated pathway scoring, co-expression networks, and machine-learning features from peripheral blood and yielded three principal findings: (i) cases exhibited quantifiably elevated “pan-PCD activity,” which was stably coupled to a specific co-expression module; (ii) PCD signals changed in parallel with a neutrophil-dominant innate immune phenotype, suggesting a putative PCD–DAMPs–NETs–BBB pathophysiological cascade; and (iii) a parsimonious three-gene panel (CREBBP, ANTXR2, ARG1) demonstrated discriminative performance in independent cohorts and aligned with established biology, thereby forming an initial bridge from mechanism to drug repurposing. Taken together, our results support the use of a multi-omics/multi-algorithm framework in peripheral blood to delineate the immune–PCD axis of AIS and provide actionable anchors for risk stratification and therapeutic development.

We first verified a global increase in “pan-PCD activity” at the peripheral blood level and linked this signal to module-level features in the co-expression network. Prior reviews and mechanistic work indicate that ischemia-induced energy failure and oxidative stress can concomitantly activate multiple PCD programs (e.g., apoptosis, ferroptosis, pyroptosis), with cross-talk and mutual amplification among pathways; these signals can reverberate in peripheral immune cells and be captured transcriptionally (Zhang L. et al., 2024; Güler et al., 2022). Methodologically, compared with single-gene differential analysis alone, sample-level pathway scoring (e.g., ssGSEA) combined with WGCNA preserves information about “network co-regulation,” mitigating both technical and biological noise; applications in stroke peripheral blood datasets have shown utility for identifying molecular features and subtypes associated with clinical outcomes (Song J. et al., 2023; Yang et al., 2022). Accordingly, the observed coupling between higher PCD pathway scores and a specific network module suggests that PCD reflects not an aggregation of isolated genes but a cross-sample, stable network reconfiguration that may index brain–periphery interactions along the inflammation–cell-death axis. It is important to acknowledge that changes in peripheral cellular composition (e.g., increased neutrophil proportions) may by themselves contribute to the elevated pan-PCD pathway scores observed in blood, since ssGSEA-based scoring on bulk peripheral-blood transcriptomes does not distinguish cell-intrinsic activation of death programs from shifts in leukocyte abundance (Stamova et al., 2026). The present blood-level findings should therefore be interpreted as a population-level transcriptional pattern rather than as direct evidence that PCD programs are activated within specific cell populations. Future work should integrate deconvolution/single-cell approaches and longitudinal sampling to disentangle composition-driven from cell-intrinsic contributions and to confirm the independence and robustness of this peripheral signal (Song Z. et al., 2023).

The neutrophil-centered immune phenotype aligns with evidence that neutrophil extracellular traps (NETs) compromise BBB integrity and are associated with worse stroke outcomes (Tu et al., 2024; He et al., 2025). Moreover, preclinical and clinical studies suggest that inhibiting NET formation or attenuating NET-related oxidative/proteolytic activity may improve BBB permeability and functional outcomes, lending support to our proposed chain model of “PCD-induced DAMPs → neutrophil recruitment/NETs → BBB disruption → secondary injury (Gu et al., 2024).”

This framework is also consistent with the temporal organization of peripheral immune responses in AIS: innate immunity predominates in the hyperacute/acute phases, while T-cell subsets (e.g., central/effector memory CD8, Th17) undergo time-dependent migration and functional remodeling, as reported in transcriptomic and immunophenotyping studies of peripheral blood (Di et al., 2025; Qiao et al., 2025). Thus, our neutrophil-skewed enrichment and the observed correlations in immune infiltration provide peripheral corroboration for the involvement of the NETs–BBB pathway in early AIS pathophysiology.

Using complementary feature-selection strategies, we identified a three-gene panel—CREBBP, ANTXR2, and ARG1—that confers diagnostic discrimination and offers clear biological interpretability. CREBBP encodes a histone acetyltransferase regulating inflammatory programs and neuronal survival (Masci et al., 2024). HDAC inhibitors such as TSA have shown neuroprotective effects in ischemic models (Lisek et al., 2025; Zhang L. Y. et al., 2024; Liao et al., 2020). In peripheral blood, CREBBP expression likely reflects systemic transcriptional responses rather than direct effector activity. ANTXR2 is linked to endothelial–basement membrane interactions and barrier homeostasis (Wu et al., 2023), making its upregulation biologically plausible. Its emergence through convergent feature selection positions it as a data-driven candidate requiring further validation. ARG1 sits at the nexus of myeloid immunometabolism, modulating the arginine–NO axis and contributing to immune suppression/resolution; recent reviews and experimental studies indicate that post-stroke upregulation of ARG1 in myeloid cells is associated with remodeling of the immune microenvironment, and excessive ARG1 activity may impede functional recovery, positioning it as both a measurable peripheral marker and a potential therapeutic target (Karadima et al., 2025; Kim et al., 2025). Drug-repurposing analyses nominated TSA as a candidate consistent with CREBBP biology. Vascular-active agents such as papaverine warrant caution given potential BBB effects (Cho et al., 2020) and require rigorous dose-timing optimization. This pathway-gene-drug framework provides tractable leads for prospective validation and mechanistic studies.

It should be noted that although CREBBP, ANTXR2, and ARG1 were consistently selected by both LASSO and SVM-RFE, their individual diagnostic performance showed notable inter-cohort variability. Specifically, CREBBP achieved a discovery-cohort AUC of 0.974 but dropped to 0.656 in the external test cohort, indicating potential overfitting or cohort-specific confounding. This discrepancy likely reflects differences in microarray platforms, patient demographics, blood collection protocols, and pre-analytical variables between the two datasets. In contrast, ANTXR2 and ARG1 maintained more robust discriminative performance across cohorts (0.981 vs. 0.931 and 0.937 vs. 0.901, respectively). These observations underscore the necessity of rigorous external validation for any single-gene biomarker and suggest that a multi-gene combinatorial model may offer greater cross-cohort stability than individual gene-level metrics alone.

Concerning ARG1, its role in stroke is mechanistically complex: while this analysis identifies ARG1 upregulation as part of the peripheral-blood PCD-associated signature, recent experimental evidence indicates that ARG1-expressing infiltrating macrophages can exert detrimental effects on post-stroke functional recovery through L-arginine depletion and local immunosuppression (Kim et al., 2025). Therefore, the observed elevation of ARG1 in peripheral blood should not be interpreted as uniformly protective; rather, it may reflect a context-dependent myeloid response that can be either adaptive or maladaptive depending on the spatiotemporal dynamics of the post-stroke immune microenvironment. Future studies with cell-type resolution and functional validation are needed to clarify whether peripheral ARG1 upregulation represents a compensatory anti-inflammatory response or contributes to stroke-induced immunosuppression.

This study has several limitations. First, reliance on public transcriptomic datasets limits control over confounders such as time-to-sampling, comorbidities, and concomitant medications, and harmonized patient-level clinical annotation (e.g., stroke severity, lesion characteristics) was not uniformly available, constraining stratified interpretation and inferences about the dynamics of the immune–PCD trajectory. Additionally, the sample size of the discovery cohort (GSE16561, n = 63) is modest, and WGCNA, LASSO, and SVM-RFE—as data-driven algorithms—may be prone to overfitting in smaller datasets. The high AUCs observed for ANTXR2 and ARG1 (0.90–0.98) warrant cautious interpretation in this context. While external validation in an independent cohort (GSE58294, n = 92) demonstrated consistent performance, both datasets are derived from similar microarray platforms. Prospective multicenter studies with larger cohorts and cross-platform validation will be valuable to further establish the generalizability and clinical utility of these three genes. Second, the pan-PCD and immune-infiltration scores were derived from bulk peripheral-blood transcriptomes via ssGSEA and cannot disentangle cell-intrinsic pathway engagement from shifts in leukocyte composition; cell-type-resolved approaches (deconvolution, single-cell RNA-seq, or orthogonal cytometry) were not part of this design. Likewise, NET formation, DAMP release, and BBB injury were not directly measured, so the proposed “PCD–DAMPs–NETs–BBB” cascade remains a working hypothesis pending dedicated experimental confirmation. Third, diagnostic performance was reported at the single-gene level and reached only moderate discrimination across cohorts; an integrated multi-gene model was not formally developed in the present study, and clinical utility will likely require such a model combined with clinical scales, neuroimaging, and routine hematologic indices. Fourth, the rat MCAO experiment was an exploratory, small-scale cross-species component without quantitative neurological scoring or histological infarct verification; brain-tissue upregulation in rats does not substitute for independent validation in human blood cohorts, which will be required before any formal biomarker claim. Fifth, the pharmacological component is largely informed by literature, gene–drug enrichment, and *in silico* docking, and remains exploratory and hypothesis-generating; vascular-active compounds warrant vigilance regarding BBB safety, and a staged biomarker-guided translational pathway will be essential before clinical deployment.

## Conclusion

We established and externally validated a peripheral-blood pan-PCD signature for ischemic stroke by quantifying pan-PCD activity with ssGSEA, integrating WGCNA and differential analysis to nominate candidates, and converging via LASSO and RFE on CREBBP, ANTXR2, and ARG1 as robust biomarkers with discernible diagnostic performance and a neutrophil-skewed immune context. *In vivo* validation in a rat MCAO model further confirmed upregulation of all three genes in the ischemic brain tissue. Drug prioritization through Enrichr/DSigDB and molecular docking nominated papaverine and TSA as computationally prioritized candidates for future preclinical evaluation, outlining an exploratory pathway–gene–drug framework. Prospective multicenter validation with higher-resolution multi-omics/single-cell profiling and rigorous assessment of PCD–immune–targeted interventions will be needed to translate these findings into clinical benefit.

## Data Availability

The human transcriptomic datasets analyzed in this study are publicly available from the Gene Expression Omnibus (GEO) under accession numbers GSE16561 (https://www.ncbi.nlm.nih.gov/geo/query/acc.cgi?acc=GSE16561) and GSE58294 (https://www.ncbi.nlm.nih.gov/geo/query/acc.cgi?acc=GSE58294). All analysis code is available from the corresponding author upon reasonable request.

## References

[B1] Alsbrook D. L. Di Napoli M. Bhatia K. Biller J. Andalib S. Hinduja A. (2023). Neuroinflammation in acute ischemic and hemorrhagic stroke. Curr. Neurol. Neurosci. Rep. 23 (8), 407–431. 10.1007/s11910-023-01282-2 37395873 PMC10544736

[B2] Amini H. Knepp B. Rodriguez F. Jickling G. C. Hull H. Carmona-Mora P. (2023). Early peripheral blood gene expression associated with good and poor 90-day ischemic stroke outcomes. J. Neuroinflammation 20 (1), 13. 10.1186/s12974-022-02680-y 36691064 PMC9869610

[B3] Blase A. di Girasole C. G. Benjamin L. Turowski P. (2025). Phased blood-brain barrier disruption in ischaemic stroke: implications for therapy? Fluids Barriers CNS 22 (1), 90. 10.1186/s12987-025-00701-5 40866935 PMC12382098

[B4] Charoentong P. Finotello F. Angelova M. Mayer C. Efremova M. Rieder D. (2017). Pan-cancer immunogenomic analyses reveal genotype-immunophenotype relationships and predictors of response to checkpoint blockade. Cell. Rep. 18 (1), 248–262. 10.1016/j.celrep.2016.12.019 28052254

[B5] Chi Q. Liang F. Zhang Y. Chen C. Chen X. Pan Y. (2025). Integrated transcriptome and single-cell RNA sequencing analysis revealed the prognostic significance of GBP4 in pancreatic adenocarcinoma. Transl. Oncol. 61, 102532. 10.1016/j.tranon.2025.102532 40944976 PMC12496431

[B6] Cho S. Ling Y. H. Lee M. J. Chen S. P. Fuh J. L. Lirng J. F. (2020). Temporal profile of blood-brain barrier breakdown in reversible cerebral vasoconstriction syndrome. Stroke 51 (5), 1451–1457. 10.1161/strokeaha.119.028656 32299322

[B7] Chou M. L. Babamale A. O. Walker T. L. Cognasse F. Blum D. Burnouf T. (2023). Blood-brain crosstalk: the roles of neutrophils, platelets, and neutrophil extracellular traps in neuropathologies. Trends Neurosci. 46 (9), 764–779. 10.1016/j.tins.2023.06.005 37500363

[B8] Di G. Vázquez-Reyes S. Díaz B. Peña-Martinez C. García-Culebras A. Cuartero M. I. (2025). Daytime DNase-I administration protects mice from ischemic stroke without inducing bleeding or tPA-Induced hemorrhagic transformation, Even with aspirin pretreatment. Stroke 56 (2), 527–532. 10.1161/strokeaha.124.049961 39869712 PMC11771350

[B9] Du S. Yang B. Wang X. Li W. Y. Lu X. H. Zheng Z. H. (2020). Identification of potential leukocyte antigen-related protein (PTP-LAR) inhibitors through 3D QSAR pharmacophore-based virtual screening and molecular dynamics simulation. J. Biomol. Struct. Dyn. 38 (14), 4232–4245. 10.1080/07391102.2019.1676825 31588870

[B10] Fang H. Bo Y. Hao Z. Mang G. Jin J. Wang H. (2024). A promising frontier: targeting NETs for stroke treatment breakthroughs. Cell. Commun. Signal 22 (1), 238. 10.1186/s12964-024-01563-4 38654328 PMC11036592

[B11] Feigin V. L. Brainin M. Norrving B. Martins S. O. Pandian J. Lindsay P. (2025). World stroke organization: global stroke fact sheet 2025. Int. J. Stroke 20 (2), 132–144. 10.1177/17474930241308142 39635884 PMC11786524

[B12] Gao M. Ran Y. Qi J. Han X. Wei Y. Wang K. (2026). Integrating single-cell and spatial transcriptomics to dissect mast-cell heterogeneity and arginine-metabolism-associated markers in BRCA. Neoplasia 73, 101281. 10.1016/j.neo.2026.101281 41655499 PMC12907235

[B39] GBD 2021 Stroke Risk Factor Collaborators (2024). Global, regional, and national burden of stroke and its risk factors, 1990-2021: a systematic analysis for the global burden of disease study 2021. Lancet Neurol. 23 (10), 973–1003. 10.1016/S1474-4422(24)00369-7 39304265 PMC12254192

[B13] Gong Z. Guo J. Liu B. Guo Y. Cheng C. Jiang Y. (2023). Mechanisms of immune response and cell death in ischemic stroke and their regulation by natural compounds. Front. Immunol. 14, 1287857. 10.3389/fimmu.2023.1287857 38274789 PMC10808662

[B14] Gu X. Dong M. Xia S. Li H. Bao X. Cao X. (2024). γ-Glutamylcysteine ameliorates blood-brain barrier permeability and neutrophil extracellular traps formation after ischemic stroke by modulating Wnt/β-catenin signalling in mice. Eur. J. Pharmacol. 969, 176409. 10.1016/j.ejphar.2024.176409 38365105

[B15] Güler M. C. Tanyeli A. Ekinci Akdemir F. N. Eraslan E. Özbek Şebin S. Güzel Erdoğan D. (2022). An overview of ischemia-reperfusion injury: review on oxidative stress and inflammatory response. Eurasian J. Med. 54 (1), 62–65. 10.5152/eurasianjmed.2022.22293 36655447 PMC11163358

[B16] Guo Z. Liu Y. Chen D. Sun Y. Li D. Meng Y. (2025). Targeting regulated cell death: apoptosis, necroptosis, pyroptosis, ferroptosis, and cuproptosis in anticancer immunity. J. Transl. Int. Med. 13 (1), 10–32. 10.1515/jtim-2025-0004 40115032 PMC11921819

[B17] Han Y. Yu X. Yin Y. Lv Z. Jia C. Liao Y. (2021). Identification of potential BRAF inhibitor joint therapy targets in PTC based on WGCAN and DCGA. J. Cancer 12 (6), 1779–1791. 10.7150/jca.51551 33613767 PMC7890315

[B18] Hänzelmann S. Castelo R. Guinney J. (2013). GSVA: gene set variation analysis for microarray and RNA-seq data. BMC Bioinforma. 14, 7. 10.1186/1471-2105-14-7 23323831 PMC3618321

[B19] He W. Wu Z. Liu Y. Ye Z. (2025). Neutrophil extracellular traps in ischemic stroke: mechanisms, clinical implications, and therapeutic potential. Front. Neurol. 16, 1641985. 10.3389/fneur.2025.1641985 41018178 PMC12463598

[B20] Hong L. Zhu J. He Z. Wang X. Li S. Liu X. (2025). Effect of time delay on reperfusion after tenecteplase in an extended time window: analysis from the CHABLIS-T trials. J. Am. Heart Assoc. 14 (12), e040994. 10.1161/jaha.124.040994 40497495 PMC12229177

[B21] Huang T. Kou L. Zhang Q. Liu X. Hu X. (2025). Single-cell RNA sequencing reveals palmitoylation-driven cellular heterogeneity and prognostic biomarkers in lung adenocarcinoma. Transl. Oncol. 61, 102501. 10.1016/j.tranon.2025.102501 40811979 PMC12363589

[B22] Karadima E. Chavakis T. Alexaki V. I. (2025). Arginine metabolism in myeloid cells in health and disease. Semin. Immunopathol. 47 (1), 11. 10.1007/s00281-025-01038-9 39863828 PMC11762783

[B23] Kim H. S. Jee S. A. Einisadr A. Seo Y. Seo H. G. Jang B. S. (2025). Detrimental influence of Arginase-1 in infiltrating macrophages on poststroke functional recovery and inflammatory milieu. Proc. Natl. Acad. Sci. U. S. A. 122 (7), e2413484122. 10.1073/pnas.2413484122 39951507 PMC11848331

[B24] Liao Y. Cheng J. Kong X. Li S. Li X. Zhang M. (2020). HDAC3 inhibition ameliorates ischemia/reperfusion-induced brain injury by regulating the microglial cGAS-STING pathway. Theranostics 10 (21), 9644–9662. 10.7150/thno.47651 32863951 PMC7449914

[B25] Lisek M. Bochenska N. Tomczak J. Duraj J. Boczek T. (2025). Epigenetic regulation in ischemic neuroprotection: the dual role of HDACs and HATs in neuroinflammation and recovery. Antioxidants (Basel) 14 (8), 1015. 10.3390/antiox14081015 40867911 PMC12382868

[B26] Liu S. Wang Z. Zhu R. Wang F. Cheng Y. Liu Y. (2021). Three differential expression analysis methods for RNA sequencing: limma, EdgeR, DESeq2. J. Vis. Exp. 175. 10.3791/62528 34605806

[B27] Liu J. Bai C. Yang H. Song L. Xu H. Sun Y. (2025). Machine learning-driven identification of blood-based biomarkers and therapeutic agents for personalized ischemic stroke management. J. Cardiovasc. Transl. Res. 18 (4), 924–940. 10.1007/s12265-025-10635-w 40694178 PMC12436529

[B28] Majumder D. (2024). Ischemic stroke: pathophysiology and evolving treatment approaches. Neurosci. Insights 19, 26331055241292600. 10.1177/26331055241292600 39444789 PMC11497522

[B29] Martin S. S. Aday A. W. Almarzooq Z. I. Anderson C. A. M. Arora P. Avery C. L. (2024). 2024 heart disease and stroke statistics: a report of US and global data from the American heart association. Circulation 149 (8), e347–e913. 10.1161/cir.0000000000001209 38264914 PMC12146881

[B30] Masci D. Puxeddu M. Silvestri R. La Regina G. (2024). Targeting CBP and p300: emerging anticancer agents. Molecules 29 (19), 4524. 10.3390/molecules29194524 39407454 PMC11482477

[B31] Qi Z. Zhu L. Wang K. Wang N. (2023). PANoptosis: emerging mechanisms and disease implications. Life Sci. 333, 122158. 10.1016/j.lfs.2023.122158 37806654

[B32] Qian S. Long Y. Tan G. Li X. Xiang B. Tao Y. (2020). Programmed cell death: molecular mechanisms, biological functions, diseases, and therapeutic targets. MedComm 5 (12), e70024. 10.1002/mco2.70024 PMC11604731 39619229

[B33] Qiao S. Yuan J. Zhang S. C. Lu Y. Y. Zhou P. Xin T. (2025). Neutrophil extracellular traps in central nervous system disorders: mechanisms, implications, and emerging perspective. Front. Immunol. 16, 1602336. 10.3389/fimmu.2025.1602336 40469282 PMC12133456

[B34] Qin C. Yang S. Chu Y. H. Zhang H. Pang X. W. Chen L. (2022). Signaling pathways involved in ischemic stroke: molecular mechanisms and therapeutic interventions. Signal Transduct. Target Ther. 7 (1), 215. 10.1038/s41392-022-01064-1 35794095 PMC9259607

[B35] Rust R. (2023). Ischemic stroke-related gene expression profiles across species: a meta-analysis. J. Inflamm. (Lond). 20 (1), 21. 10.1186/s12950-023-00346-x 37337154 PMC10280959

[B36] Song J. Zaidi S. A. A. He L. Zhang S. Zhou G. (2023a). Integrative analysis of machine learning and molecule docking simulations for ischemic stroke diagnosis and therapy. Molecules 28 (23), 7704. 10.3390/molecules28237704 38067435 PMC10707570

[B37] Song Z. Yu J. Wang M. Shen W. Wang C. Lu T. (2023b). CHDTEPDB: transcriptome expression profile database and interactive analysis platform for congenital heart disease. Struct. Congenit. Heart Dis. 18 (6), 693–701. 10.32604/chd.2024.048081

[B38] Stamova B. Knepp B. Rodriguez F. (2026). Molecular heterogeneity in human stroke - what can we learn from the peripheral blood transcriptome? J. Cereb. Blood Flow. Metab. 46 (6), 1424–1446. 10.1177/0271678x251322598 40079561 PMC11907527

[B40] Tay J. K. Narasimhan B. Hastie T. (2023). Elastic net regularization paths for all generalized linear models. J. Stat. Softw. 106, 1. 10.18637/jss.v106.i01 37138589 PMC10153598

[B41] Tripathi S. K. Muttineni R. Singh S. K. (2013). Extra precision docking, free energy calculation and molecular dynamics simulation studies of CDK2 inhibitors. J. Theor. Biol. 334, 87–100. 10.1016/j.jtbi.2013.05.014 23727278

[B42] Tu H. Ren H. Jiang J. Shao C. Shi Y. Li P. (2024). Dying to defend: neutrophil death pathways and their implications in immunity. Adv. Sci. (Weinh) 11 (8), e2306457. 10.1002/advs.202306457 38044275 PMC10885667

[B43] Wang Q. Liu N. Simo L. Ma Q. Li C. (2025). A narrative review of reperfusion therapy in acute ischemic stroke: emerging advances, current challenges, and future directions. Brain Circ. 11 (3), 187–199. 10.4103/bc.bc_161_24 40842448 PMC12367269

[B44] Wu D. Chen Q. Chen X. Han F. Chen Z. Wang Y. (2023). The blood-brain barrier: structure, regulation, and drug delivery. Signal Transduct. Target Ther. 8 (1), 217. 10.1038/s41392-023-01481-w 37231000 PMC10212980

[B45] Xiong H. Liu J. Zhang S. Guo X. (2026). Unveiling shared PANoptosis mechanisms in LUAD and osteoarthritis *via* bioinformatics and machine learning. Transl. Oncol. 63, 102586. 10.1016/j.tranon.2025.102586 41207123 PMC12639453

[B46] Yang Z. Wang G. Luo N. Tsang C. K. Huang L. (2022). Consensus clustering of gene expression profiles in peripheral blood of acute ischemic stroke patients. Front. Neurol. 13, 937501. 10.3389/fneur.2022.937501 35989931 PMC9388856

[B47] Yang H. (2022). Comprehensive analysis of the expression and clinical significance of a ferroptosis-related genome in ovarian serous cystadenocarcinoma: a study based on TCGA data. Oncologie 24 (4), 835–863. 10.32604/oncologie.2022.026447

[B48] Zhang L. Hu Z. Li Z. Lin Y. (2024a). Crosstalk among mitophagy, pyroptosis, ferroptosis, and necroptosis in central nervous system injuries. Neural Regen. Res. 19 (8), 1660–1670. 10.4103/1673-5374.389361 38103229 PMC10960298

[B49] Zhang L. Y. Zhang S. Y. Wen R. Zhang T. N. Yang N. (2024b). Role of histone deacetylases and their inhibitors in neurological diseases. Pharmacol. Res. 208, 107410. 10.1016/j.phrs.2024.107410 39276955

[B50] Zhao Y. Ma X. Meng X. Li H. Tang Q. (2024). Integrating machine learning and single-cell transcriptomic analysis to identify potential biomarkers and analyze immune features of ischemic stroke. Sci. Rep. 14 (1), 26069. 10.1038/s41598-024-77495-3 39478056 PMC11525974

[B51] Zhao Z. Zheng Z. Jiang S. Zhang L. Tang X. (2026). Multi-omics analysis unveils tumor heterogeneity and immunotherapy predictive model in breast cancer for precision medicine and early detection. Neoplasia 71, 101260. 10.1016/j.neo.2025.101260 41344266 PMC12719570

[B52] Zou Y. Xie J. Zheng S. Liu W. Tang Y. Tian W. (2022). Leveraging diverse cell-death patterns to predict the prognosis and drug sensitivity of triple-negative breast cancer patients after surgery. Int. J. Surg. 107, 106936. 10.1016/j.ijsu.2022.106936 36341760

